# Family physicians understanding about Mantoux test: A survey from a high endemic TB country

**DOI:** 10.1186/1447-056X-9-8

**Published:** 2010-05-31

**Authors:** Niloufer Sultan Ali, Kishwar Jamal, Ali Khan Khuwaja

**Affiliations:** 1 Family Medicine, Aga Khan University, Karachi, Pakistan; 2 Family Medicine/Community Health Sciences, Aga Khan University, Karachi, Pakistan

## Abstract

**Background:**

Tuberculosis is a global health emergency and is a big challenge to diagnose and manage it. Family physicians being first contact health persons should be well competent to diagnose and manage the patients with tuberculosis.

**Aims:**

This study was aimed to assess the level of understanding about Mantoux Test amongst Family Physicians in Karachi, Pakistan and to determine the difference of level of understanding by gender and number of tuberculosis patients seen in a month.

**Methods:**

A cross sectional survey was conducted among 200 Family Physicians working in Karachi; the largest city and economic hub of Pakistan. Family Physicians who attended Continuous Medical Education sessions were approached after taking consent. Pre-tested, self administered questionnaire was filled consisting of: basic demographic characteristics, questions regarding knowledge about Mantoux Test, its application and interpretation. Data of 159 questionnaires was analyzed for percentages, as rest were incomplete. Chi square test was used to calculate the difference of understanding levels between various groups.

**Results:**

Almost two thirds of respondents were males and above 35 years of age. Majority of Family Physicians were private practitioners and seeing more than five tuberculosis patients per month. Overall, a big gap was identified about the knowledge of Mantoux Test among study participants. Only 18.8% of Family Physicians secured Excellent (≥ 80% correct responses). This poor level of understanding was almost equally distributed in all comparative groups (Male = 20.8% versus Female = 15.9%; p - 0.69) and (Seen < 5 tuberculosis patients per month = 18.6% versus seen ≥ 5 tuberculosis patients per month = 19.3%; p - 0.32). A huge majority of Family Physicians (92%) however, showed keen interest in obtaining further knowledge regarding Mantoux Test and amongst them 72% suggested Continued Medical Education sessions as preferable mode of updating themselves.

**Conclusion:**

Our study revealed an overall major deficit in understanding and interpretation of Mantoux Test amongst Family Physicians which needs to be addressed. Continues Medical Education sessions for Family Physicians should be organized in regular basis for upgrading their knowledge in this regards.

## Background

Tuberculosis (TB) was declared a global health emergency by World Health Organization (WHO) [1]. According to WHO Global TB Report, Pakistan suffers from the eighth-highest burden in the world, with a TB prevalence of 263/100,000 population and TB deaths estimated at 34/100,000 population [2]. In a survey, conducted in Karachi, Pakistan, Marsh et al has reported TB as a second leading cause of adult death [3]. The setbacks and hurdles which are encountered in tackling this disease mainly include late and improper diagnosis and management [4]. Like many countries in the developing world the public health care system in Pakistan is neither very efficient nor very accessible. However, there is a very strong private health sector, particularly in the cities and a major bulk of the population consults these private family physicians (FP's). According to an estimate 80% of TB patients in urban Pakistan initially report to private FP's for their diagnosis and treatment [5]. However research focusing on TB management by the FP's is almost non-existent. The few studies that have been conducted in Pakistan revealed that the knowledge and practices regarding diagnosis and management of TB is very unsatisfactory amongst FPs [6,7] which can leads to drug resistance and increased mortality [8].

Clinical research on the MT has shown that a diagnosis of active case of tuberculosis is never made solely on the results of this test [9]. Ali et al [10] has reported that 44% of healthy health care workers had a positive MT; out of them none developed active disease even after one year of follow up. Thus, tuberculin response in TB-endemic area can not be used as a diagnostic marker for active TB [11]. Hence; starting treatment just on the basis of positive MT test is never recommended.

Family physicians being first contact health persons should be well competent and updated to diagnose and manage the tuberculosis particularly in countries where tuberculosis is one of the major health problems. Several studies that have been conducted in Pakistan assessed the knowledge regarding TB and its management, but none of them have particularly focused on the knowledge and the interpretation of MT by the FPs. We therefore conducted this study to assess the in-depth knowledge, interpretation and application of the MT test amongst FPs in Karachi. Results of this study will help and guide to formulate and implement the interventions for FPs in this regards.

## Methods

A questionnaire-based study was conducted among FPs who attended Continuous Medical Education sessions; which were arranged specifically for FPs in Karachi, Pakistan. After taking consent to participate in the study, a self-administered questionnaire was distributed to all (200) FP's. Full confidentiality of the information gathered was ensured to all the study participants and also assured that the results of this study would not be presented on individual level. Even though, no harm was expected to occur to any of the study participant, study questionnaire and proposal was reviewed and approved by the Research Committee of the department of Family Medicine, Aga Khan University, Karachi.

Questionnaire, consisting of basic demographic characteristics about the participants and questions regarding their knowledge about MT and its application and interpretation were filled by the respondents. Face and content validity of the questionnaire was obtained through a review process with experts in the filed. After incorporating the identified inconsistencies and inaccuracies, the questionnaire was pre-tested on a group of family medicine residents (trainees) to identify any problem relating to question design, flow and interpretation. Feedback given were incorporated accordingly. A total of 10 questions were asked from the study participants and about 20 minutes were needed to respond the questionnaire completely. Each correct response was marked as one point, those who responded five to seven questions correctly were labeled as 'Good' while those who responded correctly to eight and more questions were labeled as 'Excellent'.

Data of 159 questionnaires was analyzed using the statistical software package SPSS Version 16, remaining were not included because of incompleteness of the forms. Percentages and their 95% CIs were calculated for each variable. Chi square test was used to measure the significant difference between various groups (Male vs. female and < 5 TB pts vs. ≥ 5TB pts seen by FP per month), keeping the level of significance (α) at 0.05. Out of total 159 respondents 60% were males and 73.5% were seen more than five TB patients per month.

## Results

Level of knowledge about MT amongst FPs and their knowledge differences by sex and number of TB patients seen per month are summarized in Table 1. About 30% of FP's reported the use of MT as a diagnostic tool for detection of active case of TB. More than half of the respondents did not know the correct response regarding the cutoffs for positivity of MT for both HIV and Non-HIV patients. More than two thirds of the respondents also did not know the correct possible causes of a negative MT result. Similarly, majority of responses were not correct for other questions. Overall, less than one-fifth of the study participants achieved the score of ≥ 80 and this poor knowledge was equally prevalent in all compared groups (Male = 20.8%, Female = 15.9%; p - 0.69) and (Seen < 5 TB patients per month = 18.6%, seen ≥ 5 TB patients per month = 19.3%; p - 0.32).

**Table 1:** Level of understanding about MT amongst FP's and their differences by gender and number of TB patients seen in a month

Questions	FPs responded correctly (%)	Gender (%)		p-value	Number of TB patients seen in a month (%)		p-value
					
		Male	Female		< 5	≥ 5	
Should MT be used as a diagnostic test to detect active TB?	66.6	64.6	68.3	*0.38*	64.9	68.3	*0.56*

What is the route of administration of M.T.?	73.0	67.7	78.3	*0.05*	75.3	70.7	*0.17*

How many tuberculin units should be injected?	29.6	33.3	25.9	*0.13*	29.9	29.3	*0.57*

After how many hours tuberculin reaction should be read?	62.9	66.7	59.1	*0.15*	59.8	66.0	*0.19*

How tuberculin reaction should be measured?	61.7	67.7	55.7	*0.08*	55.7	67.7	*0.02*

What is the cutoff for positivity of MT in non HIV patients?	41.5	44.8	38.2	*0.19*	34	49.0	*0.02*

What is the cutoff for positivity of MT in HIV patients?	31.4	31.1	31.7	*0.54*	28.9	33.9	*0.20*

How will you manage an asymptomatic patient with MT ≥15 mm?	50.9	57.3	44.5	*0.04*	46.4	55.4	*0.08*

Does negative MT exclude TB?	89.3	85.4	93.2	*0.04*	89.7	88.9	*0.45*

What are the causes of negative M.T.?	30.2	33.3	27.1	*0.19*	30.9	29.5	*0.26*

**Overall score**							

Good	57.9	60.4	55.4	*0.26*	51.5	64.3	*0.07*

**Figure 1:**
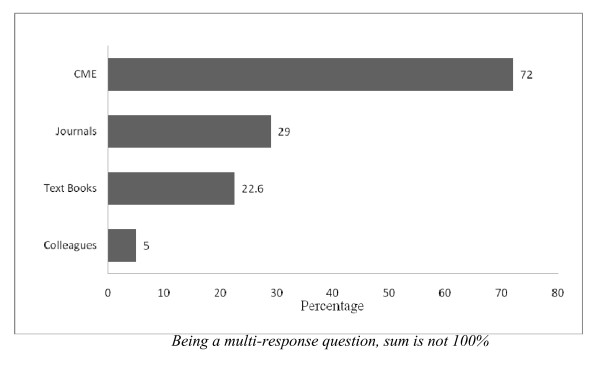
**Preference of respondents to obtain further knowledge about MT**.

Regarding the interest to seek further knowledge about the subject, almost 92% of the respondents shown their keen interest. Amongst them, 72% had preference for continuing medical education sessions (CME) for updating themselves while the second most preferred option reported as scientific medical journals and newsletters. (Figure 1).

## Discussion

To the best of our knowledge this study is so far the first of its kind conducted in high endemic country, Pakistan which specifically focused on the knowledge of MT among private FPs who are supposed to be the first contact health care providers for the community in general. This study revealed that there is an overall major deficit in the understanding and interpretation of the MT amongst FP's of either sex and irrespective of the number of TB patients they see per month.

In this study, over one-fourth of the respondents did not know the correct route of administration of MT and about one-third of FPs has reported that they use MT as a screening tool to detect active TB. Nearly three-fourths of FPs did not know the number of tuberculin units that are injected during MT and around 60% of FP's did not know that a MT reading > 10 mm is taken as positive in non-HIV patients in Pakistan. About half of the respondents said they would start anti-tuberculosis treatment on an asymptomatic patient with MT readings of 15 mm or more. About three-fourths of the respondents gave incorrect answers for the causes of negative MT and this poor understanding was equally prevalent in all comparative groups. Overall, proportions of correct responses of some of the questions were reported higher by FPs who were seeing more than five patients per month however this difference is not noteworthy.

Despite the widespread use of MT by the FPs, major gaps regarding its knowledge and clinical interpretation and application were identified. Less than one fifth of the study participants scored at the level of ≥ 80% and this low figure was equally documented among all study groups. Khan [7] and Manalo [12] also reported poor knowledge about MT among first contact health persons in high endemic TB prevalent countries.

However, it was encouraging to note that aver 90% of the FP's showed interest in gaining more knowledge about MT and its interpretation and clinical application. Almost two thirds expressed the need to obtain this information through CME.

## Conclusion

In spite of study participants practicing in urban Pakistan and attending CMEs, a big gap was identified about the understanding of MT. We can assume even more unsatisfactory knowledge about this important topic from FPs of rural and remote settings who does not have opportunity to upgrade their medical knowledge. Continuous update on the recent evidence based knowledge should be incorporated through CME sessions, lectures, seminars, workshops and hand outs and booklets. More research work at larger scale is also suggested in this important topic amongst FPs in Pakistan.

## Competing interests

The authors declare that they have no competing interests.

## Authors' contributions

NSA conceived and designed the study and prepared the manuscript. KJ developed and administered the questionnaires and managed the data. AKK analyzed and interpreted the data and provided intellectual feedback throughout the study. All authors read and approved the final manuscript.
